# Survival Rate of Short, Locking Taper Implants with a Plateau Design: A 5-Year Retrospective Study

**DOI:** 10.1155/2015/197451

**Published:** 2015-04-16

**Authors:** Kemal Özgür Demiralp, Nihat Akbulut, Sebnem Kursun, Didem Argun, Nilsun Bagis, Kaan Orhan

**Affiliations:** ^1^Ministry of Health, Public Hospitals Agency of Turkey, 06520 Ankara, Turkey; ^2^Oral and Maxillofacial Surgery Department, Faculty of Dentistry, Gaziosmanpaşa University, 60150 Tokat, Turkey; ^3^Division of Dentomaxillofacial Radiology, Ministry of Health, Bolu Oral and Dental Health Center, 14300 Bolu, Turkey; ^4^Special Clinic Practice, 06490 Ankara, Turkey; ^5^Department of Periodontology, Faculty of Dentistry, Ankara University, 06500 Ankara, Turkey; ^6^Dentomaxillofacial Radiology Department, Faculty of Dentistry, Ankara University, 06500 Ankara, Turkey

## Abstract

*Background*. Short implants have become popular in the reconstruction of jaws, especially in cases with limited bone height. Shorter implants, those with locking tapers and plateau root shapes, tend to have longer survival times. We retrospectively investigated the cumulative survival rates of Bicon short implants (<8 mm) according to patient variables over a 5-year period. *Materials and Methods*. This study included 111 consecutively treated patients with 371 implants supporting fixed or removable prosthetics. Data were evaluated to acquire cumulative survival rates according to gender, age, tobacco use, surgical procedure, bone quality, and restoration type. Statistics were performed using chi-square, Mann-Whitney, and Kruskal Wallis H tests. *Results*. The survival rate was 97.3% with, on average, 22.8 months of follow-up. Patients older than 60 years had higher failure rate than the other age groups (*P* < 0.05). Placed region, age, and bone quality had adverse effects on survival rate in the <8 mm implant group with statistically significant difference (*P* < 0.05). *Conclusions*. Approximately 23-month follow-up data indicate that short implants with locking tapers and plateau-type roots have comparable survival rates as other types of dental implants. However, due to limitations of study, these issues remain to be further investigated in future randomized controlled clinical trials.

## 1. Introduction

The use of short implants (<8 mm), especially in jaws with less bone height, has long been a source of debate and has been associated with a low success rate [1, 2]. However, the development of implant designs such as the plateau root form [3] and implants with surface structures has increased the success rate to above 90% [1].

Primary stability, which depends on surgical technique (immediate versus normal placements, stage 1 versus stage 2) and the properties of the bone in which the implant is placed (types I, II, III, and IV), is important for dental implant success [4]. In bone of poor quality, increasing implant diameter may be the only way to increase tolerance to occlusal forces, improve initial stability, and provide a favorable stress distribution to the surrounding bone [5]. Finite element analyses have also suggested that maximum bone stress is particularly related to implant design but practically independent of implant length [6]. Successful osseointegration has been documented [7], and systemic factors such as advanced age and smoking [8] may be related to failure to achieve osseointegration and wound healing [9].

The Bicon short implant system (Bicon, LLC) is a screwless implant system. The implant and implant-abutment unit connect with a 3° locking taper in a screwless system [10]. The high-friction force created by the locking taper breaks down the titanium oxide layer, and the metals become fused together in a cold weld [10]. This locking taper connection-type implant with conical abutment has the potential to develop a microbial seal [10, 11], leading to decreased peri-implant bone loss due to microbial invasion and successful osseointegration.

In the present study, we retrospectively investigated the cumulative survival rates of Bicon short implants (shorter than 8 mm) and compare them with 8 mm implants according to patient variables in a 5-year cohort study using radiological assessments during long-term follow-up.

## 2. Methods

### 2.1. Study Design and Sample

The present study was a retrospective cohort study. We retrieved patient data, including radiographs, from medical records. The cohort was composed of patients with at least one Bicon short implant (<8 mm) and (8 mm) implants placed between November 2007 and February 2012 at the Faculty of Dentistry, University of Ankara, Ankara, Turkey.

All patients provided written informed consent. Ethical approval was obtained from the Human Research Ethics Committee of our institution (clearance certificate: 13-KAEK-131).

### 2.2. Study Variables

#### 2.2.1. Demographics and Health Status

All patient data, including age, gender, and systemic conditions, were noted before the surgical procedure. Age was classified as older or younger than 60. Patients who were categorized as being medically compromised, patients with evidence of bone disease, relevant drug consumption, skeletal asymmetries or trauma, congenital disorders, and pathological disorders of the maxilla or mandible, and syndromic patients were excluded.

#### 2.2.2. Anatomical Variables

From patient files and radiographs, implant location and bone quality (types I–III; there were no type IV cases) were noted. Bone quality was ascertained clinically at the time of implant placement according to surgeon judgment. The two radiologist coauthors investigated patient panoramic X-rays (Figure 2) in terms of bone quality. All radiographs were taken with a conventional (Planmeca Proline, Helsinki, Finland) imaging unit at machine settings of 66–70 kVp, 6 mA (Eastman Kodak Co., Rochester, NY, USA), or with a digital unit (Planmeca ProMax) at 66–70 kVp, 8 mA, as recommended by the manufacturer. All postsurgical radiographs were obtained as digital panoramic radiographs. The conventional film radiographs were digitized with a flatbed scanner (Epson Expression 10000 XL) with a transparency adapter. Digitization was performed at 300 dpi and in grayscale. Digital films were stored in a computer database using the manufacturer's software (Dimaxis Pro, version 4.0.5, Planmeca).

#### 2.2.3. Implant-Specific Variables

**Figure 1:**
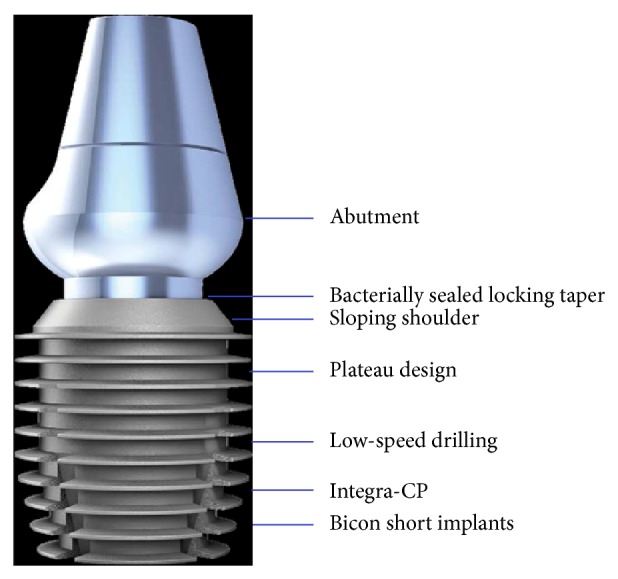
Features of the Bicon short implant.

**Figure 2:**
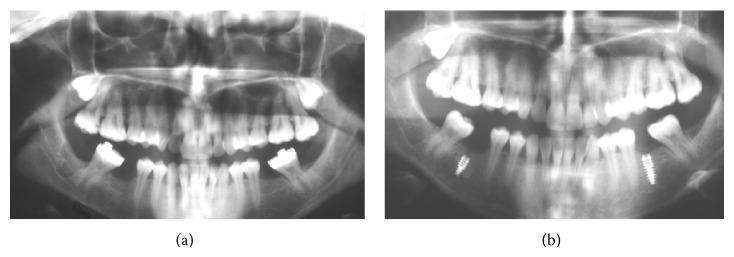
(a) Presurgical radiograph, (b) 6-month follow-up radiograph with (<8 mm) on the right and (8 mm) on the left side of the same patient.

The Bicon Integra-CP system (Bicon, LLC) was used (Figure 1). Implant-related variables included length (5–8 mm), diameter (3–5 mm), and well size (2-3 mm). It was also noted whether the surgery was a two-stage surgery or an immediate or normal surgical placement. Normal implant placements were carried out in edentulous crestal bone areas. From the patient files, we also noted whether the type of restoration for each implant was a fixed or removable (overdenture) prosthesis.

#### 2.2.4. Implant Survival Evaluation

Implant failure was the primary outcome variable. For an adequate treatment the following criteria were taken into account: clinical stability with complete function and no discomfort to the patient, no suppuration or infection present, and also no radiolucent areas around the implants in a radiographic examination [1, 7].

#### 2.2.5. Data Management and Statistics

A database was created using Excel (Microsoft), with appropriate checks made to identify errors. Then the data were transferred to the SPSS (Statistical Package for the Social Sciences) software (version 15.0) for statistical analyses. Frequency and percentage distributions were calculated. Based on the results of normality tests, when examining differences between groups, the Mann-Whitney *U* test was used in double groups with nonnormal distributions of variables. With more than two groups with nonnormal distributions of variables, the Bonferroni-corrected Kruskal-Wallis *H*-test was used. To examine dependence between variables, the chi-squared test was used. A *P* value <0.05 was considered to indicate statistical significance.

## 3. Results

In total, 111 patients were treated with 371 short Bicon implants supporting fixed and removable prostheses. The mean age of the patients was 45.7 (range, 18–67) years; 13.2% of patients were older than 60 and 86.8% were younger than 60 (Table 1). Of the patients, 47 (39.9%) were women. All patients were in good health and 28% were smokers (Table 1). Descriptive implant variables are summarized in Table 2.

### 3.1. Implant Survival Findings

The mean duration of clinical follow-up was 22.8 (range, 2–51) months after surgical placement of the implants (Table 3). In total, 10 of the 371 implants failed, giving a cumulative survival rate of 97.3% with, on average, 22.8 months of follow-up (Tables 2 and 3). All failures occurred during the healing or osseointegration phase before prosthetic loading.

Detailed statistical analyses revealed that some factors (demographic, smoking, and anatomical factors) were related with implant success. Patients with implants (<8 mm) older than 60 years had a higher failure rate than the patients under 60 years (*P* < 0.05; Table 4). However, no significant difference was found for 8 mm implants according to age groups in terms of implant failure (*P* > 0.05). Overall, again no statistical significant difference was found according to gender groups in terms of implant failure for both <8 mm and 8 mm groups (*P* > 0.05).

There was no significant difference (*P* > 0.05) between tobacco users and nonsmokers in terms of implant survival rate (Table 5) for both groups. However, surgical implant placement technique (immediate versus normal placements) significantly affected the survival rate of short implants (*P* < 0.05). Implants placed using the normal surgical technique had a higher survival rate than those placed using the immediate technique (Table 6).

In Table 7, statistical significant difference was found in implant failure between implants placed in the mandibular, premolar-molar regions. It was indicated that the implants shorter than 8 mm (<8 mm) in the mandibular premolar-molar region had higher failure rate than the others (*P* < 0.05).  Table 8 shows the implant failure rate versus bone type I, II, or III. Type II bone had higher survival rates while type III had the highest failure rate with a significant difference in implants shorter than 8 mm (<8 mm) whereas no statistical significance was found regarding bone quality (types) in terms of implant failure for 8 mm implants (*P* > 0.05). Similarly, there was no effect of prosthetic rehabilitation on implant failure rate for all implant groups (Table 9).

## 4. Discussion

After loading, all of the short implants were functional with no complications. Consistent with previous studies, all implant failures were seen in the early phases of treatment [1, 12, 13].

One of our main findings is the cumulative survival rate of 97.3% with, on average, 22.8 months of follow-up. The second major finding is the average marginal bone loss of 0.41 mm in the same follow-up time. Consistent with our results, Gentile et al. [2] reported a survival rate of 95.2% in patients with short implants during a 1-year follow-up. Vandeweghe et al. [14] reported a survival rate of 96.5% in 13 patients with short implants supporting single crowns (2 implants failed).

Short dental implants have become more popular in recent years because of increased survival and success rates due to changes in design and structural features [1–4, 9, 10, 13–17]. One of the more popular short dental implant systems is the Bicon short implant (Bicon, LLC), which has a conical, 3.0° locking taper, is screwless, and has a plateau root form. The plateau root form provides some benefits in terms of healing and osseointegration, where the healing chambers become filled with a blood clot, which is eventually replaced with new bone, leading to biomechanical fixation [15]. When this process takes place as expected, it leads to significantly improved healing patterns compared to screw root form implants [15]. Plateau designs also have greater surface areas despite being shorter, maximizing osseous contact [12]. A significant advantage of having an abutment-implant connection with a locking taper is that there are no screws that can loosen or fracture or other component failures. It also seals the margin of the abutment—crown—implant interface, which can harbor bacteria [10]. Because of these benefits, Bicon short implants (<8 mm) were used to rehabilitate the patients in the present study.

Vehemente et al. [18], using the same type of implant as in the present study, reported 12- and 60-month survival rates of 95.2% and 90.2%, respectively. Rossi et al. [19] reported a survival rate of 95% in a cohort study of 6 mm implants with 24 months of follow-up. Finally, many other studies have reported survival rates of short implants ranging from 88% to 100% [1, 4, 9, 12, 13].

In the present study, implants in patients over 60 years old had a lower survival rate (91.84%) than those in younger patients for <8 mm implants (98.14%, *P* < 0.05). These findings may be related to systemic factors that decrease vascularity or contribute to delayed wound healing, such as those seen in the elderly and smokers [8, 9]. In contrast, Gentile et al. [2] reported that the mean age of patients at implant placement did not affect the survival rate of implants.

In the present study, surgical procedures affected the survival rate. Implants that were placed using the normal surgical technique had a higher survival rate (*P* < 0.05) than those placed using the immediate surgical technique for both <8 mm and 8 mm implants. Prosthetic loadings were conducted 3–8 months after implant placement, and two-stage surgeries were the other standardized techniques used. Immediate placement of implants has several advantages: fewer surgical interventions, shorter treatment time, ideal three-dimensional implant positioning, presumptive preservation of alveolar bone at the side of tooth extraction, and soft tissue esthetic features [20]. In contrast with the present findings, most studies have suggested no difference between immediate and delayed surgical placement of short implants in terms of survival rate [20–23]. In a 2-year follow-up study by Palattella et al. [22], 16 patients received single tooth replacement with immediate implants and a control group received implants at healed sites; they did not find a statistically significant difference in marginal bone loss between the test and control groups. Other studies have reported similar results [20, 22, 24].

In the present study, survival rate factors including gender, tobacco usage, and restoration type did not affect the survival rate of implants but placed region, age, and bone quality did (*P* < 0.05). Consistent with the present findings, Kumar et al. [8] reported that smoking was not related to implant survival or the osseointegration process, especially when using surface-modified dental implants, as in this study. The fact that smoking did not affect the survival rate may have been due to the short follow-up time. Many studies have reported a relationship between smoking and osseointegration failure [8, 9]. Various reports have correlated smoking with poor-quality bone, type IV [8]. Such bone may lower the primary stability of implants. Thus, using surface-modified implants with acceptable primary stability in poorer-quality bone, such as the HA-coated implants used in this study, may be better.

We also found no statistically significant differences (*P* < 0.05) between prosthesis types in terms of survival rate of the implant especially between single crown and bridge restorations. Patients restored with crown-bridges had higher degrees of survival rate than those restored with crowns. A possible reason may be the occurrence of lateral or overload application [25]. Aglietta et al. [26] reported that single-unit crowns and short-span fixed dental prostheses with cantilever-supported implants did not show any difference in survival of the implant. Similarly, Naert et al. [27] found that the use of subsequently situated single-implant crowns to restore an edentulous space did not lead to more marginal bone loss than around splinted implants.

In conclusion, using HA-coated, short (≤8 mm), plateau-type, locking taper, screwless implants, the cumulative survival rate was 97.3% with an average follow-up of 22.83 months, a satisfactory result. However, placed region, age, and bone quality had adverse effects on survival rate. Randomized controlled clinical trials with longer follow-up should be conducted to determine the cumulative survival rate and benefits of short implants in a grander context.

## Figures and Tables

**Table 1:** Implants placed according to patient demographics.

		*n* (patient)	%	*n* (implant)	%
Gender	Male	64	57.66	223	60.11
	Female	47	42.34	148	39.89
	Total	**111**	**100**	**371**	**100**

Age	Over 60	98	88.3	322	86.79
	Under 60	13	11.7	49	13.21
	Total	**111**	**100**	**371**	**100**

Tobacco use	No	79	71.17	267	71.97
	Yes	32	28.83	104	28.03
	Total	**111**	**100**	**371**	**100**

**Table 2:** Implant variables.

		*n *	%
Length (mm)	<8 mm	118	31.81
	8 mm	253	68.19
	Total	**371**	**100**

Width (mm)	3 mm	4	1.08
	3.5 mm	76	20.49
	4 mm	62	16.71
	4.5 mm	132	35.58
	5 mm	97	26.15
	Total	**371**	**100**

Well diameter (mm)	2 mm	148	39.89
	2.5 mm	1	0.27
	3 mm	222	59.84
	Total	**371**	**100**

Implant region	Mandibular incisor region	29	7.82
	Maxillary incisor region	53	14.29
	Mandibular premolar-molar region	127	34.23
	Maxillary premolar-molar region	162	43.67
	Total	**371**	**100**

Bone quality	Type I	10	2.70
	Type II	278	74.93
	Type III	83	22.37
	Total	**371**	**100**

Surgical technique	Immediate placement	42	11.32
	Normal placement	329	88.68
	Total	**371**	**100**

Restoration type	Crown	56	15.09
	Crown-bridge	299	80.59
	Overdenture	16	4.31
	Total	**371**	**100**

Implant survival	Failed	10	2.70
	Functional	361	97.30
	Total	**371**	**100**

**Table 3:** Distribution of implant variables and follow-up time.

	*n*	Mean	Median	Min	Max	SS
Length (mm)	371	7.35	8	5	8	0.95
Width (mm)	371	4.33	4.5	3	5	0.55
Well diameter (mm)	371	2.63	3	2	3	0.47
Follow-up time after surgery (months)	371	22.83	23	2	51	10.90
Follow-up time after prosthesis (months)	371	17.41	18	0	47	10.93

**Table 4:** Implant failure distribution in terms of age group.

		Age						Statistical analysis	
Implant failure		Under 60		Over 60		Total			
		*n*	%	*n*	%	*n*	%	Chi-square test	*P*
<8 mm	Failed	2	1,9	3	27,3	5	4,2		
	Functional	105	98,1	8	72,7	113	95,8	Fisher's exact test	**0.006**
	Total	**107**	**100**	**11**	**100**	**118**	**100**		

8 mm	Failed	4	1,9	1	2,6	5	2		
	Functional	211	98,1	37	97,4	248	98	Fisher's exact test	0.56
	Total	**215**	**100**	**38**	**100**	**253**	**100**		

**Table 5:** Survival of implants according to smoking and nonsmoking groups.

		Smoking or nonsmoking						Chi-square test	
Implant failure		No		Yes		Total			
		*n*	%	*n*	%	*n*	%		
<8 mm	Failed	3	3,7	2	5,6	5	4,2		
	Functional	79	96,3	34	94,4	113	95,8	Fisher's exact test	0.64
	Total	**82**	**100**	**36**	**100**	**118**	**100**		

8 mm	Failed	3	1,6	2	2,9	5	2		
	Functional	182	98,4	66	97,1	248	98	Fisher's exact test	0.613
	Total	**185**	**100**	**68**	**100**	**253**	**100**		

**Table 6:** Survival of implants according to surgical placement technique.

		Surgical placement techniques						Statistical analysis	
Implant failure		Immediate		Normal		Total			
		*n*	%	*n*	%	*n*	%	Chi-square test	*P*
<8 mm	Failed	5	4,4	0	0	5	4,2		
	Functional	5	100	108	95,6	113	95,8	Fisher's exact test	**0.001**
	Total	**5**	**100**	**113**	**100**	**118**	**100**		

8 mm	Failed	4	10,8	1	0,5	5	2		
	Functional	33	89,2	215	99,5	248	98	Fisher's exact test	**0.002**
	Total	**37**	**100**	**216**	**100**	**253**	**100**		

**Table 7:** Implant failure distribution according to placed region.

			Implant failure						Chi-square test
			Failed		Functional		Total	
			*n*	%	*n*	%	*n*	%	*P*
<8 mm	Placed region	Mandibular incisor	0	0	3	2,7	3	2,5	**0.024**
		Maxillary incisor	0	0	3	2,7	3	2,5	
		Mandibular premolar-molar	5	100	35	31	40	33,9	
		Maxillary premolar-molar	0	0	72	63,7	72	61	
		Total	**5**	**100**	**113**	**100**	**118**	**100**	

8 mm	Placed region	Mandibular incisor	0	0	26	10,5	26	10,3	0.255
		Maxillary incisor	2	40	48	19,4	50	19,8	
		Mandibular premolar-molar	3	60	84	33,9	87	34,4	
		Maxillary premolar-molar	0	0	90	36,3	90	35,6	
		Total	**5**	**100**	**248**	**100**	**253**	**100**	

**Table 8:** Survival of implants according to bone quality.

			Implant failure						Chi-square test
			Failed		Functional		Total	
			*n*	%	*n*	%	*n*	%	*P*
<8 mm	Bone quality	Type I	0	0	1	0,9	1	0,8	**0.044**
		Type II	1	20	94	83,2	95	80,5	
		Type III	4	80	18	15,9	22	18,6	
		Total	**5**	**100**	**113**	**100**	**118**	**100**	

8 mm	Bone quality	Type I	0	0	9	3,6	9	3,6	0.25
		Type II	2	40	181	73	183	72,3	
		Type III	3	60	58	23,4	61	24,1	
		Total	**5**	**100**	**248**	**100**	**253**	**100**	

**Table 9:** Survival of implants according to prosthetic rehabilitation.

			Implant failure						Chi-square test
			Failed		Functional		Total	
			*n*	%	*n*	%	*n*	%	*P*
<8 mm	Prosthetic rehabilitation	Crown	0	0	21	18,9	21	18,9	0.99
		Crown-bridge	0	0	90	81,1	90	81,1	
		Total	**0**	**0**	**111**	**100**	**111**	**100**	

8 mm	Prosthetic rehabilitation	Crown	0	0	28	11,7	28	11,7	1
		Crown-bridge	1	100	195	81,6	196	81,7	
		Overdenture	0	0	16	6,7	16	6,7	
		Total	**1**	**100**	**239**	**100**	**240**	**100**	

## References

[1] Maló P., Nobre M. D. A., Rangert B. (2007). Short implants placed one-stage in maxillae and mandibles: a retrospective clinical study with 1 to 9 years of follow-up. *Clinical Implant Dentistry and Related Research*.

[2] Gentile M. A., Chuang S.-K., Dodson T. B. (2005). Survival estimates and risk factors for failure with 6 × 5.7-mm implants. *The International Journal of Oral & Maxillofacial Implants*.

[3] Suzuki M., Calasans-Maia M. D., Marin C. (2010). Effect of surface modifications on early bone healing around plateau root form implants: an experimental study in rabbits. *Journal of Oral and Maxillofacial Surgery*.

[4] Draenert F. G., Sagheb K., Baumgardt K., Kämmerer P. W. (2012). Retrospective analysis of survival rates and marginal bone loss on short implants in the mandible. *Clinical Oral Implants Research*.

[5] Kido H., Schulz E. E., Kumar A., Lozada J., Saha S. (1997). Implant diameter and bone density: effect on initial stability and pull-out resistance. *The Journal of Oral Implantology*.

[6] Pierrisnard L., Renouard F., Renault P., Barquins M. (2003). Influence of implant length and bicortical anchorage on implant stres distribution. *Clinical Implant Dentistry and Related Research*.

[7] Adell R., Lekholm U., Rockler B., Brånemark P. I. (1981). A 15-year study of osseointegrated implants in the treatment of the edentulous jaw. *International Journal of Oral Surgery*.

[8] Kumar A., Jaffin R. A., Berman C. (2002). The effect of smoking on achieving osseointegration of surface-modified implants: a clinical report. *International Journal of Oral and Maxillofacial Implants*.

[9] Pieri F., Aldini N. N., Fini M., Marchetti C., Corinaldesi G. (2012). Preliminary 2-year report on treatment outcomes for 6-mm-long implants in posterior atrophic mandibles. *The International Journal of Prosthodontics*.

[10] Urdaneta R. A., Marincola M., Weed M., Chuang S.-K. (2008). A screwless and cementless technique for the restoration of single-tooth implants: a retrospective cohort study. *Journal of Prosthodontics*.

[11] Dibart S., Warbington M., Su M. F., Skobe Z. (2005). In vitro evaluation of the implant-abutment bacterial seal: the locking taper system. *International Journal of Oral and Maxillofacial Implants*.

[12] Goené R., Bianchesi C., Hüerzeler M. (2005). Performance of short implants in partial restorations: 3-year follow-up of Osseotite implants. *Implant Dentistry*.

[13] Lops D., Bressan E., Pisoni G., Cea N., Corazza B., Romeo E. (2012). Short implants in partially edentulous maxillae and mandibles: a 10 to 20 years retrospective evaluation. *International Journal of Dentistry*.

[14] Vandeweghe S., de Ferrerre R., Tschakaloff A., de Bruyn H. (2011). A wide-body implant as an alternative for sinus lift or bone grafting. *Journal of Oral and Maxillofacial Surgery*.

[15] Coelho P. G., Suzuki M., Guimaraes M. V. M. (2010). Early bone healing around different implant bulk designs and surgical techniques: a study in dogs. *Clinical Implant Dentistry and Related Research*.

[16] Urdaneta R. A., Marincola M. (2007). The integrated abutment Crown, a screwless and cementless restoration for single-tooth implants: a report on a new technique: techniques and technologies. *Journal of Prosthodontics*.

[17] Urdaneta R. A., Leary J., Lubelski W., Emanuel K. M., Chuang S.-K. (2012). The effect of implant size 5 × 8 mm on crestal bone levels around single-tooth implants. *Journal of Periodontology*.

[18] Vehemente V. A., Chuang S.-K., Daher S., Muftu A., Dodson T. B. (2002). Risk factors affecting dental implant survival.. *The Journal of oral implantology*.

[19] Rossi F., Ricci E., Marchetti C., Lang N. P., Botticelli D. (2010). Early loading of single crowns supported by 6-mm-long implants with a moderately rough surface: a prospective 2-year follow-up cohort study. *Clinical Oral Implants Research*.

[20] Ortega-Martínez J., Pérez-Pascual T., Mareque-Bueno S., Hernández-Alfaro F., Ferrés-Padró E. (2012). Immediate implants following tooth extraction. A systematic review. *Medicina Oral, Patologia Oral y Cirugia Bucal*.

[21] Annibali S., Cristalli M. P., Dell'Aquila D., Bignozzi I., La Monaca G., Pilloni A. (2012). Short dental implants: a systematic review. *Journal of Dental Research*.

[22] Palattella P., Torsello F., Cordaro L. (2008). Two-year prospective clinical comparison of immediate replacement vs. immediate restoration of single tooth in the esthetic zone. *Clinical Oral Implants Research*.

[23] Schropp L., Kostopoulos L., Wenzel A., Isidor F. (2005). Clinical and radiographic performance of delayed-immediate single-tooth implant placement associated with peri-implant bone defects. A 2-year prospective, controlled, randomized follow-up report. *Journal of Clinical Periodontology*.

[24] Lindeboom J. A. H., Tjiook Y., Kroon F. H. M. (2006). Immediate placement of implants in periapical infected sites: a prospective randomized study in 50 patients. *Oral Surgery, Oral Medicine, Oral Pathology, Oral Radiology and Endodontology*.

[25] Akça K., Iplikcioğlu H. (2002). Finite element stress analysis of the effect of short implant usage in place of cantilever extensions in mandibular posterior edentulism. *Journal of Oral Rehabilitation*.

[26] Aglietta M., Iorio Siciliano V., Blasi A. (2012). Clinical and radiographic changes at implants supporting single-unit crowns (SCs) and fixed dental prostheses (FDPs) with one cantilever extension. A retrospective study. *Clinical Oral Implants Research*.

[27] Naert I., Koutsikakis G., Quirynen M., Duyck J., van Steenberghe D., Jacobs R. (2002). Biologic outcome of implant-supported restorations in the treatment of partial edentulism. Part 2: a longitudinal radiographic evaluation. *Clinical Oral Implants Research*.

